# Neutrophil-to-Lymphocyte Ratio, a Novel Inflammatory Marker, as a Predictor of Bipolar Type in Depressed Patients: A Quest for Biological Markers

**DOI:** 10.3390/jcm10091924

**Published:** 2021-04-29

**Authors:** Vlad Dionisie, Gabriela Adriana Filip, Mihnea Costin Manea, Robert Constantin Movileanu, Emanuel Moisa, Mirela Manea, Sorin Riga, Adela Magdalena Ciobanu

**Affiliations:** 1 Department of Psychiatry and Psychology, ‘Carol Davila’ University of Medicine and Pharmacy, 020021 Bucharest, Romania; mirelamanea2003@yahoo.com; 2 Department of Psychiatry, ‘Prof. Dr. Alexandru Obregia’ Clinical Hospital of Psychiatry, 041914 Bucharest, Romania; dr.robertmovileanu@gmail.com (R.C.M.); adela.ciobanu@yahoo.com (A.M.C.); 3 Department of Physiology, ‘Iuliu Hatieganu’ University of Medicine and Pharmacy, 400006 Cluj-Napoca, Romania; adrianafilip33@yahoo.com; 4 Department of Orthopaedics, Anaesthesia and Intensive Care Medicine, ‘Carol Davila’ University of Medicine and Pharmacy, 020021 Bucharest, Romania; emanuelmoisa@gmail.com; 5 ‘Elias’ University Emergency Hospital, 011461 Bucharest, Romania; 6 Department of Stress Research and Prophylaxis, ‘Prof. Dr. Alexandru Obregia’ Clinical Hospital of Psychiatry, 041914 Bucharest, Romania; D_S_Riga@yahoo.com; 7 Romanian Academy of Medical Sciences, 927180 Bucharest, Romania; 8 Neuroscience Department, Discipline of Psychiatry, ‘Carol Davila’ University of Medicine and Pharmacy, 020021 Bucharest, Romania

**Keywords:** bipolar, major depression, mania, mood, inflammation, neutrophil, lymphocyte, monocyte, systemic immune inflammatory index, biomarker

## Abstract

(1) Background: Recent research suggests inflammation as a factor involved in the pathophysiology of mood disorders. Neutrophil-to-lymphocyte (NLR), monocyte-to-lymphocyte (MLR), platelet-to-lymphocyte (PLR), and systemic immune-inflammatory (SII) index ratios have been studied as peripheral markers of inflammation in bipolar and major depressive disorders. The purpose of this study is to comparatively analyze these inflammatory ratios among manic episodes of bipolar disorder, bipolar depression and unipolar depression. (2) Methods: 182 patients were retrospectively included in the study and divided into three groups: 65 manic patients, 34 patients with bipolar depression, and 83 unipolar depressive patients. White blood cells, neutrophils, monocytes, lymphocytes, and platelets were retrieved from the patients’ database. NLR, MLR, PLR, and SII index were calculated using these parameters. (3) Results: Patients with manic episodes had elevated NLR (*p* < 0.001), MLR (*p* < 0.01), PLR (*p* < 0.05), and SII index (*p* < 0.001) compared to unipolar depression and increased NLR (*p* < 0.05) and SII index (*p* < 0.05) when compared to bipolar depression. NLR (*p* < 0.01) and SII index (*p* < 0.05) were higher in the bipolar depression than unipolar depression. NLR is an independent predictor of the bipolar type of depression in depressive patients. (4) Conclusions: The results confirm the role of inflammation in the pathophysiology of mood disorders and suggest the ability of NLR as a marker for the differentiation of bipolar from unipolar depression.

## 1. Introduction

Bipolar disorder (BD) and major depressive disorder (MDD) are common chronic psychiatric illnesses, with a prevalence in the general population of 1–1.1% and 16.2%, respectively [1,2,3]. BD is defined by mood fluctuations that range from mania or hypomania to depression and periods of euthymia, while MDD patients experience only depressive episodes [4]. BD and MDD are characterized by a recurrent course and involve a high economic burden to society [1,2,3]. Also, BD and MDD play a major contribution to the impact on the work and social life of the patients. According to results from the World Health Organisation’s World Mental Health surveys, MDD has the greatest effect on days out of role followed by BD [5]. 

Affective disorders have multiple and intricate pathophysiology pathways, thus the aetiology of affective disorders is dominated by some level of uncertainty [2,6]. Among hypothalamic–pituitary–adrenal axis dysregulation, monoamine and glutaminergic neurotransmission alterations or neurotrophins imbalance, inflammation and immune dysfunctions have been proposed as possible underlying pathways in mood disorders, both MDD and BD [2,7,8,9].

Neutrophils are cells implicated in the innate immunity. They are nonspecific inflammatory cells with phagocytic and apoptotic roles mediated by releasing the cytokines and other inflammatory molecules [10,11]. On the other hand, lymphocytes represent an important protective component of the adaptive immune response [10]. Platelets are involved in coagulation and store various pro-inflammatory molecules that modulate the immune and inflammatory response [12]. Moreover, platelets contain a large amount of serotonin that is secreted under activation conditions such as inflammation. Serotonin in turn stimulates lymphocytes, neutrophils, and monocytes, thus influencing the cytokine release. Platelets have been suggested to be involved in neuroinflammation and they have been used as peripheral models of receptor-mediated signal transduction in the central nervous system [12,13]. Monocytes are a subset of leucocytes which promote immune protection and exhibit proinflammatory effects even to drive inflammatory diseases. Monocytes have been recently in the spotlight of central nervous system pathologies since evidence shows that circulating monocyte-derived macrophage and dendritic cells could be trafficked to the central nervous system in chronic neuroinflammatory conditions where they exert a pro-inflammatory role [14,15,16,17]. Data from several studies links the activation of monocytes to the pathophysiology of various psychiatric disorders, including MDD and BD [18].

In recent years, neutrophil-to-lymphocyte ratio (NLR), platelet-to-lymphocyte ratio (PLR), and monocyte-to-lymphocyte ratio (MLR) have been studied as inflammatory markers in various diseases with chronic subclinical inflammation such as diabetes mellitus, obesity, cardio-vascular diseases, and cancers [19,20,21,22]. An increased count of neutrophils reflects the intensity of inflammatory response and a decreased count of lymphocytes represents the immune system impairment [23]. These ratios are low-cost and readily available parameters that can be calculated from routine complete blood count [24]. In addition, several papers have reported that NLR was correlated with other markers of inflammation, such as C Reactive Protein (CRP) and IL (interleukin)-6, or with oxidative stress markers (i.e., total oxidative status, total thiol) [25,26,27,28]. NLR, PLR and MLR have been studied in psychiatric disorders, including MDD and BD [24]. Numerous studies have established NLR and PLR as biomarkers for the manic phase of bipolar disorder [29,30,31,32,33,34,35]. These results have been confirmed by a meta-analysis conducted by Mazza et al. (2018). Moreover, Mazza et al. (2018) showed that only patients in the manic phase and not those in euthymia have significantly increased NLR and PLR [36]. Regarding MLR, increased levels were observed in the manic phase of bipolar illness [30,31,34]. Concerning unipolar depression, Mazza et al. (2018) also confirmed that NLR increased in MDD compared with healthy controls [36]. NLR seems to be correlated with the disease severity in unipolar depressed patients although research data presents contradictory results on this subject [37,38,39,40].

The systemic immune-inflammatory (SII) index is an integrated marker of systemic inflammation and it results from a combination of NLR and PLR. The SII index has been already extensively assessed as a marker of prognosis and mortality of patients with solid tumors or coronary artery disease [41,42,43]. Until now, very limited data is available concerning the relationship between SII index and psychiatric disorders. Wang et al. (2021) found that high SII index levels are associated with unipolar depression in male diabetic patients [44]. Also, Zhou et al. (2020) reported that MDD patients have an increased SII index compared with healthy controls [45]. A recent study showed that the degree of reduction of SII index between admission and discharge predicted the remission of depression in Coronavirus Disease (COVID) 19 survivors [46].

Based on these data, the present study aims to compare the blood cell counts, NLR, PLR, MLR, and SII index in bipolar depressive patients, bipolar manic patients, and MDD patients. Also, our objective was to explore the potential role of trait markers for these subclinical inflammation markers for bipolar or unipolar depression.

## 2. Materials and Methods

A retrospective, cross-sectional study on adult patients admitted between March 2020 and January 2021 to the psychiatric inpatient units of ”Prof. Dr. Alexandru Obregia” Clinical Hospital of Psychiatry, a tertiary centre in Bucharest (Romania), with the diagnosis of Bipolar Affective Disorder (manic and depressive episodes) or Major Depressive Disorder was conducted. The study was approved by the local Ethics Committee (decision no. 48/2021) and performed in accordance with the Declaration of Helsinki. All patients were assessed by licenced psychiatrists and the diagnosis was made based on the 10th revision of International Classification of Diseases (ICD-10) criteria. We applied the following inclusion criteria: (a) age between 18–65 years old; and (b) diagnosis of Bipolar Disorder current manic or depressive episode (F31.1–F31.5) or current episode of Major Depressive Disorder (F32.2, F32.3, F33.2, F33.3). The exclusion criteria consisted of: (a) other psychiatric comorbidities, including alcohol or other substance dependence; (b) medical conditions that could determine abnormal inflammatory parameters levels (i.e., infection; fever; endocrinological, inflammatory, autoimmune, or cerebrovascular diseases; renal, cardiac, or hepatic failure; cancer; and diabetes mellitus); (c) body mass index (BMI) > 30 kg/m^2^, heavy smoking (more than 20 cigarettes/day), pregnancy; and (d) treatment during the last two weeks prior to admission with non-steroidal anti-inflammatory drugs, aspirin, corticosteroid or immunosuppressive medication, or antibiotics.

For each patient we retrieved socio-demographic, personal and clinical features: age, gender, educational level, marital status, BMI, smoking status, and the presence of psychotic symptoms. Also, we collected data regarding biochemical and hemogram tests: white blood cells, neutrophils, lymphocytes, monocytes, platelets, fasting glycemia, and total cholesterol and triglycerides. All data were collected from each patient’s paper-based and electronic file from our hospital’s database. The NLR, PLR, MLR, and SII index were calculated using the following formulas: NLR = neutrophils count/lymphocytes count, PLR = platelets count/lymphocytes count, MLR = monocytes count/lymphocytes count, and SII index = (platelets count × neutrophils count)/lymphocytes count. All the parameters were determined from blood samples taken in the morning between 7:00 and 9:00 a.m., after fasting, in one of the first three days of hospitalisation. The blood was taken from a forearm vein and the samples were processed and analysed in the medical laboratory of “Prof. Dr. Alexandru Obregia” Clinical Hospital of Psychiatry using an automated hematology analyser (Sysmex XN-550) and an automated chemistry analyser (Architect C4000). All included patients were under psychiatric pharmacological treatment at the moment of blood sampling.

Statistical analysis was performed using the IBM Statistical Package for Social Sciences (SPSS) version 20.0 and GraphPad Prism version 8 software for Windows. Statistical analysis was performed after each variable was tested for normality of data distribution (Kolmogorov-Smirnov test). Continuous variables were expressed as mean and standard deviation. Categorical variables were expressed as percentage or frequency and standard deviation (SD). For variables with normal distribution, parametric tests were used (*T* test for independent variables, Chi-square test and Pearson correlation, and One-Way ANOVA), while for variables with non-normal distribution we used nonparametric tests (Mann-Whitney U, Kruskal Wallis). Also, the cohort was divided into three subgroups (mania, bipolar depression, and MDD). One-Way ANOVA and Kruskal Wallis analysis were used to assess the statistical differences between the three groups for continuous variables depending on data distribution. Mann-Whitney U and independent *T* test were used when we compared continuous data between two groups. Statistical analysis for categorical variables was assessed performing the Chi-square test with Pearson correlation after crosstabs were computed. For all tests, statistical significance was considered for a *p* value < 0.05 (two-tailed). For hematological indices significantly correlated with depression subtypes (Mann-Whitney U test) we performed a binary logistic regression. After univariate analysis, multivariate analysis was performed for NLR and SII Index, adjusting them for age, gender, BMI, smoking, marital status, educational status, cholesterol, glycemia, and triglycerides. Exp(B) value represents the odds ratio value at a 95% confidence interval (C.I). Statistical significance was considered for a *p* value < 0.05 (two-tailed).

## 3. Results

One-hundred and eighty-two patients (*N* = 182) met the inclusion and exclusion criteria and were included in our study. The total sample was divided into three groups as follows: 65 (35.71%) BD manic patients, 35 (19.23%) BD patients with depression, and 83 (45.6%) MDD patients. The mean (±SD) age of the sample was 44.41 (±12.29) years, with no significant difference between the number of men and women included (84 and 98, respectively). Also, more than half of the patients had between 9 to 12 years of education (51.1%) and had no psychotic symptoms (64.8%). The mean value (±SD) for BMI was 24.92 (±2.86) kg/m^2^. Other socio-demographic and clinical characteristics are presented in Table 1.

Fasting glycemia had a mean value of 92.03 ± 11.78 in the total sample. The mean value for the total cholesterol and triglycerides was 189.42 ± 45.80 and 131.0 ± 79.90, respectively, with significant difference between the three groups with regard to total cholesterol (*p* < 0.05) (Table 2).

Patients with manic episodes had higher neutrophils (5.13 ± 1.63) when compared to bipolar depressed patients (4.40 ± 1.68) (*p* < 0.01) or unipolar depressive patients (3.68 ± 1.24) (*p* < 0.001). Also, neutrophils were significantly higher in bipolar depression individuals than MDD ones (4.40 ± 1.68 vs. 3.68 ± 1.24, *p* < 0.05). In the MDD subgroup, lymphocytes were increased when compared to BD manic and BD depression subgroup, respectively (2.54 ± 0.77 vs. 2.25 ± 0.67, *p* < 0.05), (2.54 ± 0.77 vs. 2.24 ± 0.63, *p* < 0.05). Other laboratory findings and the comparisons between groups in regard to hematological parameters analysed are presented in Table 2 and Table 3, respectively.

**Figure 1:**
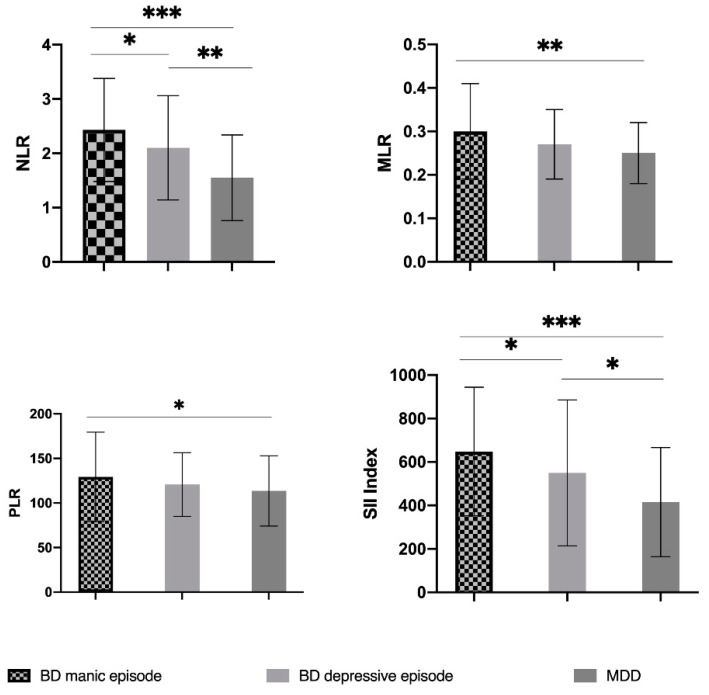
Neutrophil-to-lymphocyte ratio, platelet-to-lymphocyte ratio, monocyte-to-lymphocyte ratio, and systemic immune-inflammatory index levels in bipolar disorder patients with manic episode, bipolar disorder patients with depressive episode and patients with major depressive disorder and the comparison of ratios between groups (representation of mean ± standard deviation). The statistical significance was evaluated with Mann-Whitney U test. *, *p* < 0.05; **, *p* < 0.01; ***, *p* < 0.001; NLR, neutrophil-to-lymphocyte ratio; PLR, platelet-to-lymphocyte; MLR, platelet-to-lymphocyte; SII, systemic immune-inflammatory; BD, bipolar disorder; and MDD, major depressive disorder.

NLR was significantly increased in the BD manic group (2.43 ± 0.95) when compared to BD depression (2.10 ± 0.96) (*p* < 0.05) and MDD (1.55 ± 0.79) (*p* < 0.001). Furthermore, patients with bipolar type of depression had higher NLR than unipolar depressed subjects (2.10 ± 0.96 vs. 1.55 ± 0.79, *p* < 0.01) (Table 2 and Table 3, Figure 1). 

Both PLR and MLR were higher in patients with mania episodes than patients with unipolar depression (129.21 ± 50.19 vs. 113.5 ± 39.47, *p* < 0.05), (0.30 ± 0.11 vs. 0.25 ± 0.07, *p* < 0.01). No other significant differences were found regarding PLR or MLR (Table 2 and Table 3, Figure 1).

In patients having a manic episode of bipolar illness SII index was increased compared to individuals in the depressed phase of BD or unipolar depression (648.21 ± 295.79 vs. 549.40 ± 335.78, *p* < 0.05), (648.21 ± 295.79 vs. 415.13 ± 251.45, *p* < 0.001). Also, bipolar patients with depression (549.40 ± 335.78) have a higher SII index than unipolar depressive patients (415.13 ± 251.45) (*p* < 0.05) (Table 2 and Table 3, Figure 1).

Binary logistic regression was performed for hematological indices significantly associated with depression subtypes. The disease was considered as the dependent variable (categorical, bipolar versus unipolar depression). NLR, SII Index, age, gender, BMI, smoking, marital status, educational status, cholesterol, glycemia, and triglycerides were considered as the independent variables. Firstly, we conducted the univariate analysis for NLR and SII Index and both were significantly associated with bipolar depression (R^2^ Nagelkerke = 0.108, *p* = 0.002, OR = 2.178; 95% CI; *p* = 0.01 for NLR and R^2^ Nagelkerke = 0.059, *p* = 0.026, OR = 1.002; CI 95%, *p* = 0.047) (Table 4). Secondly, we performed multivariate analysis models adjusting NLR and SII Index for possible confounders (age, gender, BMI, smoking, marital status, educational status, total cholesterol, glycemia, and triglycerides). For the Model 2 (Table 4) we adjusted NLR for the abovementioned possible confounders (R^2^ Nagelkerke = 0.235, *p* = 0.021). After adjustment, NLR was significantly associated with bipolar depression (OR = 2.495; 95% CI; *p* = 0.005). The same adjustment for confounders made for SII Index resulted in a model that was not statistically significant (R^2^ Nagelkerke = 0.193, *p* = 0.075). For Model 3, we introduced in the same model NLR and SII Index and made the adjustment for the same confounders (R^2^ Nagelkerke = 0.248, *p* = 0.022) (Table 4). SII Index was not statistically associated with bipolar depression (OR = 0.997; 95% CI; *p* = 0.269), but NLR was associated with bipolar depression (OR = 5.311: 95% CI; *p* = 0.03) making it an independent predictor for bipolar depression (Table 4). For these binary logistic regression models, all the variables were introduced in the analysis using the Enter method.

## 4. Discussion

Growing evidence supports the involvement of immune and inflammatory pathways in the pathogenesis of mood disorders. Our study demonstrated that bipolar manic patients had an increased inflammatory state, quantified by NLR, MLR, PLR, and SII index, compared with unipolar depressed patients. Moreover, our study reported that the manic phase in BD was characterised by a higher inflammation activation, reflected by the NLR and SII index, than the depression phase. Also, NLR and SII index were increased during bipolar depression in comparison with unipolar depression. Moreover, our study reported NLR as an independent predictor for bipolar depression in patients with depressive episodes. As far as we know, this is the first study that has investigated the SII index between different mood phases in BD (i.e., mania and depression) and between patients with manic or depressive episode of BD and MDD patients. Also, this is the first research that reports NLR as a predictor of bipolar depression. Moreover, our study is the first in Romania that analysed these parameters.

Data from several meta-analyses outlines that patients suffering MDD or BD have increased different pro-inflammatory cytokines levels as compared with healthy controls [47,48,49,50]. Randomised control studies on treatment with anti-inflammatory drugs added additional support for the role of immune dysregulation and inflammation in mood disorders [51,52,53,54]. More precisely, different anti-inflammatory agents (i.e., celecoxib, statins, and omega-3 fatty acids) in monotherapy, or as an add-on to a selective serotonin reuptake inhibitor, were reported to reduce depression in patients with MDD compared with monotherapy with a selective serotonin reuptake inhibitor [51,52]. Moreover, N-acetylcysteine had an anti-depressant effect in bipolar depression as concluded by a meta-analysis conducted by Rosenblat et al. (2016) [53]. On the other hand, a recent systematic review concluded that N-acetylcysteine showed contrasting effects in bipolar depression and only some trials reported beneficial results [55]. Interestingly, celecoxib and N-acetylcysteine seemed to have some anti-manic proprieties [54,55]. Furthermore, MDD and BD are associated with different comorbidities in which inflammation is involved, including diabetes, obesity, cardiovascular disease, inflammatory bowel disease, or rheumatic diseases [56,57,58,59,60]. Treatment with interferon-based therapies can induce neuropsychiatric symptoms, including mania and most frequently depression [61]. Therefore, all these data may offer the support for the immune dysregulation and inflammation as pathogenetic mechanisms in mood disorders.

Research literature regarding NLR, MLR, and PLR between different episodes of BD and MDD showed some differences across studies. Mazza et al. (2018) were the first to compare these hemogram-derived inflammatory ratios between different phases of BD (i.e., mania and depression) [62]. They reported increased NLR and MLR values in manic patients versus bipolar depressed individuals but no differences between these two groups regarding PLR [62]. Conversely, Inanli et al. (2019) showed that only MLR is increased in manic episodes versus depression in BD [34]. On the other hand, a recent study observed that all ratios (NLR, MLR, and PLR) were increased in mania compared to bipolar depression but it is worth mentioning that the studied sample was not controlled for possible major confounders such as pathological conditions or treatment with anti-inflammatory drugs [63]. Our results are in agreement with those in the literature regarding NLR, MLR, and PLR between mania and bipolar depression. More precisely, we demonstrated that NLR, as a marker of inflammation, is increased in mania in comparison with depression of bipolar illness. In addition, Mazza et al. (2018) compared the ratios between BD mania and MDD and reported higher NLR and MLR in manic versus MDD patients [62]. Our study confirmed this data. In addition, we reported increased PLR in favor of manic episodes of BD when compared to unipolar depression. 

There is little evidence concerning SII index in different affective disorders. Only one study assessed the SII index in unipolar depression and showed that patients suffering from MDD have increased SII index levels compared with healthy controls [45]. Our study revealed, for the first time, that manic patients have a higher inflammatory state, quantified by the SII index, than bipolar depressed patients or MDD patients. These findings are in accordance with results from recent meta-analyses that reported the high levels of other inflammatory markers, such as CRP or high sensitivity-CRP, IL-6, soluble tumor necrosis factor receptor (sTNFR) 1 during the manic shift of BD compared to those with bipolar depression [64,65]. In addition, some differences were reported in the literature between BD and MDD patients. CRP, sTNFR1, IL-6 and its soluble receptor and monocyte chemoattractant protein-1 (MCP-1) were found to be higher in BD in comparison with unipolar depression [66,67,68].

Bipolar depression and unipolar depression share similar clinical presentation. Besides some clinical possible indicators of bipolarity in a patient with depressive episodes (e.g., a family history of bipolar disorder, seasonality, early onset, mood reactivity, mixed states, and switching on antidepressants), there are no other clinical or biological specific markers that could help to establish a clear diagnosis. Therefore, distinguishing bipolar from unipolar depression remains a clinical challenge [1]. Moreover, bipolar patients spend the majority of their symptomatic periods of time in the depressive phase and only 20% of bipolar patients in a depressive episode are diagnosed correctly within the first year of searching for treatment. An important aspect is that bipolar depression is frequently misdiagnosed with MDD [1,4,69]. Also, treatment between these illnesses differs substantially and prescription of antidepressants to bipolar patients is controversial and could worsen the clinical picture [70]. These problems have urged the researchers to find possible markers that support a more accurate differential diagnosis between the bipolar illness depression phase and unipolar depression. 

Regarding potential biomarkers, brain-derived neurotrophic factor was suggested to differentiate bipolar from unipolar depression and a possible cut-off value was proposed [71]. A study conducted by Brunoni et al. (2020) found that there was a different immune profile between unipolar and bipolar depression. More precisely, MDD had increased IL-1β, tumor necrosis factor α (TNF α), sTNFR 1, IL-12 and IL-10 while BD depression was characterised by an increase in IL-6, sTNFR 2, IL-18, IL-33, ST2, and KLOTHO [72]. Also, baseline CRP levels (>621.6 ng/mL) have been suggested to differentiate between bipolar and unipolar depression [73]. Another study investigated several inflammatory cytokines in BD depression and MDD. The results showed that BD depressed patients had lower TNF α and IL-13 levels than unipolar depressive patients during active episodes. After remission of depression, patients with BD had elevated IL-4 and TNF [74]. Wollenhaupt-Aguiar et al. (2020) showed that the most relevant predictor markers to differentiate bipolar from unipolar depression were IL-10, thiobarbituric acid reactive substances, and IL-4 [75]. 

In line with previous reports that outlined differences between bipolar and unipolar depression in terms of inflammation, our study found that BD depressed patients had an increased subclinical inflammation, as reflected by the SII index and NLR, when compared with MDD patients. Also, our study demonstrated that NLR is a good predictor of bipolar depression in patients with depressive episodes. More precisely, depressed patients with higher NLR values have a 5.31 increased chance to have bipolar depression. Therefore, NLR could potentially serve as an indicator of a BD illness in a depressed patient. Our results suggest that NLR could be a biomarker of an inflammatory state characteristic of the bipolar type of depression. Moreover, given their low-cost and fast availability nature, these markers could be very useful in daily practice. Of course, future prospective studies on larger samples that replicate these findings and assess them together with other inflammatory markers (i.e., CRP, IL-6) are necessary in order to draw some conclusive statements.

Our study has, however, some limitations. First of all, the study was conducted in a retrospective manner thus we could not assess the patients using a structured clinical interview. Secondly, we did not evaluate the symptoms’ severity, thus we could not analyse the possible correlation between the severity of the mood episode and ratios. Thirdly, we did not include euthymic and healthy subjects, therefore, no causal modelling is possible between BD or MDD and the NLR, MLR, PLR, and SII index. Moreover, although we controlled the results for some possible factors such as BMI, smoking status, age and gender, glycemia, and total cholesterol and triglycerides, our study lacks an assessment of other factors (i.e., lifestyle habits, menstrual cycle, and psychotropic treatment). On the other hand, a major strength of our study is that we applied exclusion criteria that helped us to control the sample for relevant confounding conditions that affect inflammation (diabetes mellitus, infection, treatment with anti-inflammatory drugs, etc.).

## 5. Conclusions

In the present study, we report important data regarding NLR, MLR, PLR, and the SII index, as inflammatory markers, and bipolar disorder and major depressive disorders. The results presented herein demonstrated that the inflammatory activation, viewed through NLR, MLR, PLR, and SII index, was higher during the manic phase of the bipolar disorder in comparison with the depressive phase of BD or unipolar depression. Also, this is the first study to report these differences regarding the SII index. Moreover, we reported altered NLR and SII index during the depressive episodes of bipolar disorder rather than that of unipolar depression. The novelty of our study is that we demonstrated that NLR is a predictor of the bipolar type of depression. These findings suggest that NLR could be proposed as a risk factor for bipolar depression in depressed patients. In addition, our results supported the hypothesis that the inflammatory response was modulated by the type of mood episode. However, further studies that assess the time relationship of these changes and their correlation with psychopathology and with other inflammatory markers are necessary to consolidate the real clinical role of these inflammatory ratios.

## Figures and Tables

**Table 1:** Socio-demographic and clinical characteristics of the patients.

	Total Sample (*N* = 182)	BD Manic Episode (*N* = 65)	BD Depressive Episode (*N* = 34)	MDD (*N* = 83)	*p*
Gender, *N* (%)					0.925 ^1^
Males	84 (46.2)	31 (47.7)	16 (47.1)	37 (44.6)	
Females	98 (53.8)	34 (52.3)	18 (52.9)	46 (56.4)	
Age (years), mean ± SD	44.41 ± 12.29	41.25 ± 11.6	47.59 ± 12.02	45.58 ± 12.45	**0.015** ^2^
Level of education, *N* (%)					**0.024** ^2^
≤8 years	38 (20.9)	7 (10.8)	7 (20.6)	24 (28.9)	
9–12 years	93 (51.1)	35 (53.8)	17 (50.0)	41 (49.4)	
>12 years	51 (28)	23 (35.4)	10 (29.4)	18 (21.7)	
Marital status, *N* (%)					0.062 ^2^
Single	68 (37.4)	35 (53.8)	10 (29.4)	23 (27.7)	
Married	62 (34.1)	15 (23.1)	12 (35.3)	35 (42.2)	
Divorced	26 (14.3)	6 (9.2)	8 (23.5)	12 (14.5)	
Widowed	6 (3.3)	0	0	6 (7.2)	
Domestic Partnership	20 (11.0)	9 (13.8)	4 (11.8)	7 (8.4)	
Smoking status, *N* (%)					**0.011 ^1^**
Yes	105 (57.7)	47 (72.3)	18 (52.9)	40 (48.2)	
No	77 (42.3)	18 (27.7)	16 (47.1)	43 (51.8)	
Psychotic symptoms, *N* (%)					**<0.001** ^1^
Yes	64 (35.2)	43 (66.2)	28 (82.4)	15 (18.1)	
No	118 (64.8)	22 (33.8)	6 (17.6)	68 (81.9)	
BMI (kg/m^2^), mean ± SD	24.92 ± 2.86	25.17 ± 2.56	25.47 ± 2.25	24.51 ± 3.25	0.449 ^2^

^1^ Chi-square test; ^2^ Kruskal Wallis test; BD, bipolar disorder; MDD, major depressive disorder; N, number of patients; BMI, body mass index; Bold values are statistically significant results.

**Table 2:** Laboratory findings (values are presented as mean ± SD).

	Total Sample	BD Manic Episode	BD Depressive Episode	MDD	*p*
White blood cells (10^3^ cells/mm^3^)	7.5 ± 1.87	8.13 ± 2	7.38 ± 1.78	7.05 ± 1.66	**0.002** ^1^
Neutrophils (10^3^ cells/mm^3^)	4.34 ± 1.61	5.13 ± 1.63	4.40 ± 1.68	3.68 ± 1.24	**0.000** ^2^
Lymphocytes (10^3^ cells/mm^3^)	2.38 ± 0.73	2.25 ± 0.67	2.24 ± 0.63	2.54 ± 0.77	**0.022** ^2^
Monocytes (10^3^ cells/mm^3^)	0.61 ± 0.17	0.64 ± 0.18	0.57 ± 0.13	0.61 ± 0.18	0.072 ^2^
Platelets (10^3^ cells/mm^3^)	264.76 ± 59.05	264.65 ± 52.26	255.35 ± 52.93	268.7 ± 65.63	0.683 ^2^
NLR	1.97 ± 0.97	2.43 ± 0.95	2.10 ± 0.96	1.55 ± 0.79	**0.000** ^2^
PLR	120.47 ± 43.56	129.21 ± 50.19	120.78 ± 35.66	113.5 ± 39.47	0.068 ^2^
MLR	0.27 ± 0.09	0.30 ± 0.11	0.27 ± 0.08	0.25 ± 0.07	**0.003** ^2^
SII Index	523.53 ± 303.58	648.21 ± 295.79	549.40 ± 335.78	415.13 ± 251.45	**0.000** ^2^
Glycemia (mg/dL)	92.03 ± 11.78	91.58 ± 12.16	94.5 ± 12.32	91.06 ± 11.41	0.158 ^1^
Total Cholesterol (mg/dL)	189.42 ± 45.80	177.18 ± 47.49	193.32 ± 49.30	197.40 ± 41.26	**0.007** ^2^
Triglycerides (mg/dL)	131.0 ± 79.90	138.40 ± 101.95	135.68 ± 78.41	123.29 ± 58.25	0.919 ^2^

^1^ ANOVA test; ^2^ Kruskal Wallis test; BD, bipolar disorder; MDD, major depressive disorder; NLR, neutrophil to lymphocyte ratio; PLR, platelet to lymphocyte ratio; MLR, monocyte to lymphocyte ratio; and SII, systemic immune-inflammatory; Bold values are statistically significant results.

**Table 3:** Comparison of laboratory variables and ratios between groups.

	BD Mania vs. BD Depression	BD Mania vs. MDD	BD Depression vs. MDD
White blood cells (10^3^ cells/mm^3^) ^1^	0.072	**<0.001**	0.344
Neutrophils (10^3^ cells/mm^3^) ^2^	**0.009**	**<0.001**	**0.038**
Lymphocytes (10^3^ cells/mm^3^) ^2^	0.754	**0.030**	**0.016**
Monocytes (10^3^ cells/mm^3^) ^2^	**0.023**	0.126	0.368
Platelets (10^3^ cells/mm^3^) ^2^	0.470	0.951	0.391
NLR ^2^	**0.047**	**<0.001**	**0.001**
PLR ^2^	0.635	**0.034**	0.113
MLR ^2^	0.095	**0.001**	0.368
SII index ^2^	**0.033**	**<0.001**	**0.015**

^1^ Student’s *t* test; ^2^ Mann-Whitney U test; BD, bipolar disorder; MDD, major depressive disorder; NLR, neutrophil to lymphocyte ratio; PLR, platelet to lymphocyte ratio; MLR, monocyte to lymphocyte ratio; and SII, systemic immune-inflammatory; Bold values are statistically significant results.

**Table 4:** Uni and multivariate analysis for NLR and SII index.

**Binary logistic regression: NLR Univariate analysis, R^2^ Nagelkerke = 0.108, *p* = 0.002**								
	B	S.E.	Wald	df	Sig.	Exp(B)	95% C.I. for Exp(B)	
							Lower	Upper
	0.778	0.301	6.692	1	0.010	2.178	1.208	3.927
**Binary logistic regression: SII Index Univariate analysis, R^2^ Nagelkerke = 0.059, *p* = 0.026**								
	B	S.E.	Wald	df	Sig.	Exp(B)	95% C.I. for Exp(B)	
							Lower	Upper
SII Index	0.002	0.001	3.950	1	0.047	1.002	1.000	1.003
**Binary logistic regression: Multivariate analysis, Model 2, R^2^ Nagelkerke = 0.235, *p* = 0.021, Method: Enter**								
	B	S.E.	Wald	df	Sig.	Exp(B)	95% C.I. for Exp(B)	
							Lower	Upper
NLR	0.914	0.328	7.780	1	0.005	2.495	1.312	4.744
Age	0.032	0.022	2.204	1	0.138	1.033	0.990	1.078
Gender	0.077	0.478	0.026	1	0.872	1.080	0.423	2.753
BMI	0.172	0.092	3.520	1	0.061	1.188	0.992	1.421
Smoking status	−0.350	0.482	0.527	1	0.468	0.705	0.274	1.812
Glycemia	0.019	0.020	0.926	1	0.336	1.020	0.980	1.061
Total Cholesterol	−0.014	0.006	4.552	1	0.033	0.987	0.974	.999
Triglycerides	0.005	0.004	1.597	1	0.206	1.005	0.997	1.012
Level of education	0.601	0.341	3.104	1	0.078	1.824	0.935	3.559
Marital status	0.141	0.199	0.503	1	0.478	1.151	0.780	1.699
Constant	−9.503	3.461	7.540	1	0.006	0.000		
**Binary logistic regression: Multivariate analysis, Model 3, R^2^ Nagelkerke = 0.248, *p* = 0.022, Method: Enter**								
	B	S.E.	Wald	df	Sig.	Exp(B)	95% C.I. for Exp(B)	
							Lower	Upper
NLR	1.670	0.768	4.730	1	0.030	5.311	1.179	23.919
SII Index	−0.003	0.002	1.221	1	0.269	0.997	0.993	1.002
Age	0.035	0.022	2.455	1	0.117	1.035	0.991	1.081
Gender	0.112	0.482	0.054	1	0.816	1.119	0.435	2.877
Smoking status	−0.202	0.502	0.162	1	0.687	0.817	0.306	2.184
BMI	0.171	0.093	3.423	1	0.064	1.187	0.990	1.423
Glycemia	0.024	0.021	1.342	1	0.247	1.025	0.983	1.068
Total Cholesterol	−0.012	0.006	3.630	1	0.057	0.988	0.975	1.000
Triglycerides	0.004	0.004	1.094	1	0.295	1.004	0.996	1.012
Level of Education	0.544	0.345	2.493	1	0.114	1.723	0.877	3.385
Marital status	0.162	0.202	0.648	1	0.421	1.176	0.792	1.747
Constant	−10.33	3.607	8.206	1	0.004	0.000		

## Data Availability

The data presented in this study are available on request from the corresponding authors. The data are not publicly available due to ethical and institutional reasons.
